# A proteomic tool suggests reductions in dementia risk following intentional weight loss in type 2 diabetes

**DOI:** 10.1002/alz70861_108444

**Published:** 2025-12-23

**Authors:** Brendan Epstein, Jessica Chadwick, Meredith Carpenter, Stephen A Williams, Shahrad Taheri, Odette Chagoury, Roy Taylor, Naveed Sattar, Michael Lean, Paul Welsh, Clare Paterson

**Affiliations:** ^1^ Standard BioTools, Boulder, CO USA; ^2^ Weill Cornell Medicine, Doha Qatar; ^3^ Hamad Medical Corporations, Doha Qatar; ^4^ Translational and Clinical Research Institute, Newcastle University, Newcastle upon Tyne UK; ^5^ Schools of Medicine and Cardiovascular and Metabolic Health, University of Glasgow, Glasgow UK

## Abstract

**Background:**

Intentional weight loss improves cardiometabolic risk factors, which by associated neuroprotective effects may reduce dementia risk. However, assessing change in dementia risk directly demands long‐term follow‐up. In this study, we assessed whether a recently validated 25‐protein signature for dementia risk could detect changes in dementia risk in response to an intentional weight loss program in people with type 2 diabetes (T2D) and associated cardiometabolic features.

**Methods:**

A potential impact of intentional weight loss on dementia risk was explored in two randomized controlled clinical trials in patients with obesity and T2D, DiRECT and DIADEM‐I; both demonstrated that a diet program to induce and maintain weight loss led to diabetes remissions of 46% and 61% at 1 year. The proteomic dementia risk test (derived from the SomaScan™ assay, predicting the probability of incident dementia diagnosis within 20‐years of blood draw) was applied to plasma samples at baseline and 1‐year in intervention (DiRECT n=118; DIADEM‐I n=56) and control (DiRECT n=144; DIADEM‐I n=66) participants. This model includes measurements of 25 plasma proteins, but not HbA1c, which might otherwise dominate a prediction of dementia risk in people with T2D. Dementia risk scores were compared within and between trial arms and the association between weight loss amount and the magnitude of dementia risk score change was explored.

**Results:**

In both DiRECT and DIADEM‐I a significant difference in the change in dementia risk score from baseline to 1‐year between the trial arms was observed (*p* =1.8x10^‐7^ and *p* =8.79x10^‐8,^ respectively), with risk scores either remaining stable or decreasing in the intervention arm over the trial duration. Furthermore, in DiRECT there was a significant (*p* =9.06x10^‐3^) association between the amount of weight lost and the reduction in dementia risk in the intervention arm with greater weight loss associating with a greater reduction in dementia risk.

**Conclusions:**

These results support epidemiological evidence that non‐pharmacological weight loss may protect against dementia risk, particularly in those with cardiometabolic features. Furthermore, the proteomic dementia risk test was robust to identifying changes in dementia risk in response to intentional weight loss. These findings suggest that the proteomic model has value to evaluate pharmacologic and non‐pharmacological interventions to reduce dementia.

